# Multivalent glycoligands with lectin/enzyme dual specificity: self-deliverable glycosidase regulators†

**DOI:** 10.1039/c9cc06376e

**Published:** 2019-10-01

**Authors:** Manuel González-Cuesta, David Goyard, Eiji Nanba, Katsumi Higaki, José M. García Fernández, Olivier Renaudet, Carmen Ortiz Mellet

**Affiliations:** a Department of Organic Chemistry, Faculty of Chemistry, University of Seville C/Profesor García González 1 41012 Seville Spain mellet@us.es; b Université Grenoble Alpes, CNRS, DCM UMR 5250 3800 Grenoble France olivier.renaudet@univ-grenoble-alpes.fr; c Organization for Research Initiative and Promotion, Tottori University 86 Nishi-cho Yonago 683-8503 Japan; d Instituto de Investigaciones Químicas (IIQ), CSIC – Universidad de Sevilla Avda. Américo Vespucio 49 Isla de la Cartuja 41092 Sevilla Spain jogarcia@iiq.csic.es; e Institut Universitaire de France 103 Boulevard Saint-Michel 75005 Paris France

## Abstract

Multivalent mannosides with inherent macrophage recognition abilities, built on β-cyclodextrin, RAFT cyclopeptide or peptide dendrimer cores, trigger selective inhibition of lysosomal β-glucocerebrosidase or α-mannosidase depending on valency and topology, offering new opportunities in multitargeted drug design.

## Abstract 2

Multimannosides assembled onto cyclodextrin or cyclopeptide cores exhibit dual specificity towards the macrophage mannose receptor and lysosomal glycosidases.

## Graphical abstract

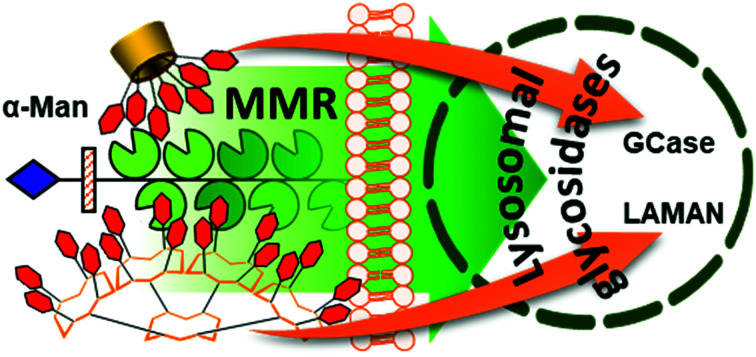

Multivalency is an underpinning action principle in the interaction of oligosaccharide moieties in glycoproteins and glycolipids with carbohydrate binding receptors (lectins) in nature.^1^ The thermodynamic and kinetic advantage of multiple glycotope moieties interacting with many receptor sites, termed ‘multivalent or cluster effect’, is attributed to several possible factors that frequently act concertedly, including steric stabilization, chelate effect, secondary subsite involvement, local concentration and cross-linking effects. Reproducing this strategy with synthetic multivalent glycoligands has proven a very effective approach for advancing our understanding on the molecular mechanisms of many biological events^2^ and also provides broad opportunities for therapeutic interventions.^3^ The external sugar coating in such devices is generally considered to impart biocompatibility and warrant a “safe” profile. Notwithstanding, recent evidence suggests that multivalency has the potential, not only to enhance the avidity towards a range of lectins sharing the same cognate sugar ligand, but also to elicit off-target inhibition of glycosidases, which may result in unforeseen promiscuity. First characterized for multimeric iminosugar-type displays,^4,5^ the scope of multivalent inhibition of carbohydrate processing enzymes has been shown to expand more generally to glycoside-exposing glycomaterials.^6^ For instance, the mannosyl-coated nanodiamond and C_60_ fullerene conjugates 1^7^ and 2^8^ (Fig. 1) were found to concurrently bind to the “matching” mannose specific lectin concanavalin A (ConA) and to inhibit the “mismatching” α-glucosidases maltase and isomaltase (Fig. 2A) as well as α- and β-galactosidases, with inhibition constant (*K*_i_) values in the 0.9–21 μM range. Surprisingly, neither 1 nor 2 were substrates for the “matching” α-mannosidase, behaving instead as weak inhibitors of the enzyme (*K*_i_ 295–320 μM). These data call for a careful evaluation of the potential risks derived from multivalency-associated “biological messiness”,^9^ expanding from the group of carbohydrate binding receptors to the category of carbohydrate processing enzymes. The change in the conceptualization of multivalency from a “natural” strategy to achieve useful responses in carbohydrate-mediated processes to a “multichannel switch” with the potential to activate/deactivate a range of receptor/enzyme recognition events is disturbing. Yet, it must be underlined that many protein receptors and enzymes are conspicuously promiscuous themselves and that promiscuity is as biologically important as specificity.^10–12^ On the other hand, the new evidences also inform the possibility of purposely conceiving glycoligands with the ability to specifically interacting with biomedically relevant lectin/glycosidase subsets, offering unprecedented opportunities in multitargeted drug design. One can conceive, for instance, that glycoarchitectures with an appropriate display of glycotopes targeting simultaneously an active endocytosis-mediating receptor and a therapeutically relevant enzyme will warrant their site-specific delivery to the target cells with no need of a third carrier device.

**Fig. 1:**
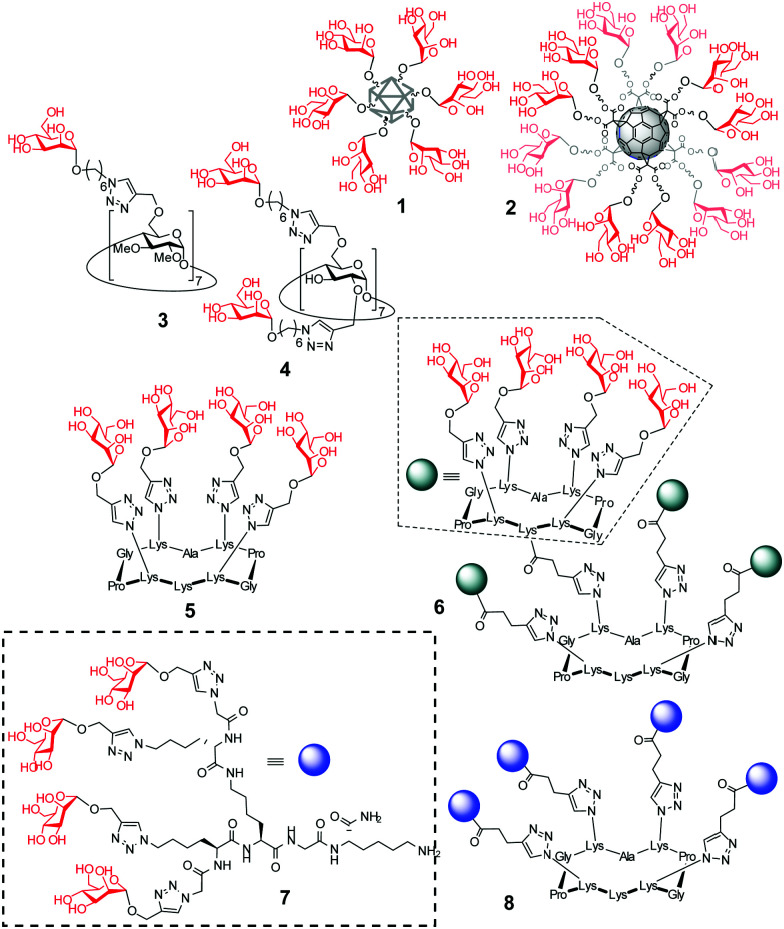
Schematic representations of the mannosylated nanodiamond and fullerene conjugates 1 and 2 previously reported to exhibit glycosidase inhibitory properties and structures of the macrophage mannose receptor (MMR)/lysosomal glycosidase dual-targeted multivalent mannosides 3–8 assessed in this work.

**Fig. 2:**
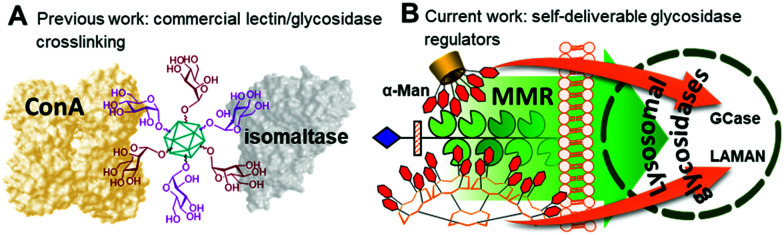
Schematic representations of the lectin/glycosidase crosslinking phenomena previously observed with glyconanodiamonds (A) and the synergistic interactions of the self-deliverable glycosidase regulators proposed in this work with the MMR membrane lectin and the therapeutically relevant glycosidases GCase or LAMAN (B).

Amalgamating cell membrane lectin-mediated uptake and glycosidase activity regulation properties in a single multivalent glycoconjugate is particularly appealing in the context of the lysosomal storage disorders (LSDs).^13^ Most patients suffering from a LSD express a mutant glycosidase that does not fold properly and undergoes endoplasmic reticulum-associated degradation. Consequently, the corresponding substrate accumulates, especially in macrophages, given rise to a range of pathologies.^13^ The potential of glycomimetic-based multivalent inhibitors to rescue the mutant enzyme, restore trafficking and enhance activity, thereby behaving as pharmacological chaperones, has been already shown for lysosomal β-glucosidase (β-glucocerebrosidase, GCase)^14^ and lysosomal α-mannosidase (LAMAN).^15^ These glycosidases are dysfunctional in Gaucher's disease and α-mannosidosis, respectively. However, the delivery and cell permeability issues commonly associated to macromolecules is an anticipated hurdle for these channels, which can be overcome by endowing the system with targeting abilities towards the macrophage mannose receptor (MMR) lectin.^16^ We hypothesized that a suitable spatial arrangement of mannosyl ligands could accrue the maximum benefit of multivalency in a combined MMR/lysosomal glycosidase context (Fig. 2B). As a proof of concept, we report here a strategy wherein multimannosides with MMR/GCase or MMR/LAMAN dual-specificity have been obtained by using β-cyclodextrin (βCD),^17^ regioselectively addressable functionalized template (RAFT) cyclopeptides^18^ and peptide dendrimers (PDs)^19^ as extrinsic scaffolds, namely compounds 3–8 (Fig. 1).

High tunability was considered as an important prerequisite in this work: several lines of evidence support that the valency and architecture of multimeric ligands can drastically affect not only the affinity but also the selectivity towards glycosidases.^20^ The ensemble of derivatives 3–8, accessible through versatile precision chemistry sequences (see the ESI† for details),^21^ was purposely defined to cover a large topological space without unduly incrementing the experimental cost.

Preliminary evidence on the feasibility of achieving disparate glycosidase inhibition selectivity with the multivalent glycoclusters 3–8 and, at the same time, retaining intrinsic lectin binding properties was achieved by conducting control experiments using both commercial glycosidases and lectins. The glycosidase panel included α- and β-glucosidases (*S. cerevisiae* and bovine liver), α- and β-galactosidases (green coffee beans and *E. coli*) and α- and β-mannosidases (jack beans and *H. pomatia*). Prior to kinetic determination of the corresponding inhibition constant (*K*_i_) values (Table S1 and Fig. S9–S18, ESI†), the enzymatic stability was investigated by gas chromatography. Free mannose was only detected in the α-mannosidase incubates (37 °C, 24 h) containing the tetravalent RAFT cyclopeptide (5) and PD (7) conjugates. In all the other cases the α-mannopyranoside linkages remained stable, meaning that any effect observed in the enzyme activity should be ascribed to an inhibitor and not to a substrate behavior. The results revealed remarkable differences in the selectivity and potency of inhibition dependent on the conjugate architecture. In general terms, the lower-valency conjugate within each pair of compounds sharing the same central scaffold, (3, 5 or 7) behaved as selective competitive inhibitor of the mammalian β-glucosidase (Glcase). The heptavalent βCD derivative 3 (*K*_i_ 7.3 μM) showed the highest inhibition potency in the series. Glcase inhibition was cancelled for the higher-valent homologues (4, 6 or 8, respectively); instead, 4–6 behaved as selective competitive inhibitors of α-mannosidase (Manase), with the hexadecavalent RAFT cyclopeptide 6 (*K*_i_ 32 μM) as the more potent representative. Enzyme-linked lectin assay (ELLA) experiments run in parallel showed that compounds 3–8 all bind ConA, a model mannose specific lectin,^2,22–24^ but not the galactose-specific peanut agglutinin (PNA), confirming the expected discrimination capacity between cognate and non-cognate lectin partners (Fig. S19, ESI†).

To substantiate the applicability of the lectin/glycosidase multispecificity concept for the title goal, we subsequently screened the ensemble of glycoclusters 3–8 as inhibitors of the acid α-glucosidase, GCase, LAMAN and β-mannosidase in cell (fibroblast) lysates. The lysosomal glycosidase inhibition selectivity pattern was qualitatively similar to that encountered against the commercial enzymes, but revealed significant differences in quantitative terms, showing exacerbated discrimination capabilities (Fig. 3A). Thus, the βCD heptamannoside 3 was a strong and selective GCase inhibitor (IC_50_ 0.1 μM, GCase/lysosomal α-glucosidase anomeric selectivity ratio over 100; Fig. S20, ESI†). The tetravalent PD conjugate 7 was also a potent inhibitor of GCase (IC_50_ 8 μM), much more efficient as compared with the macrocyclic analogue 5 (98 μM). The tetradecavalent βCD derivative 4 and the RAFT cyclopeptide hexadecamannoside 6 behaved instead as potent and highly selective LAMAN inhibitors (IC_50_ 11 and 1.0 μM, respectively).

**Fig. 3:**
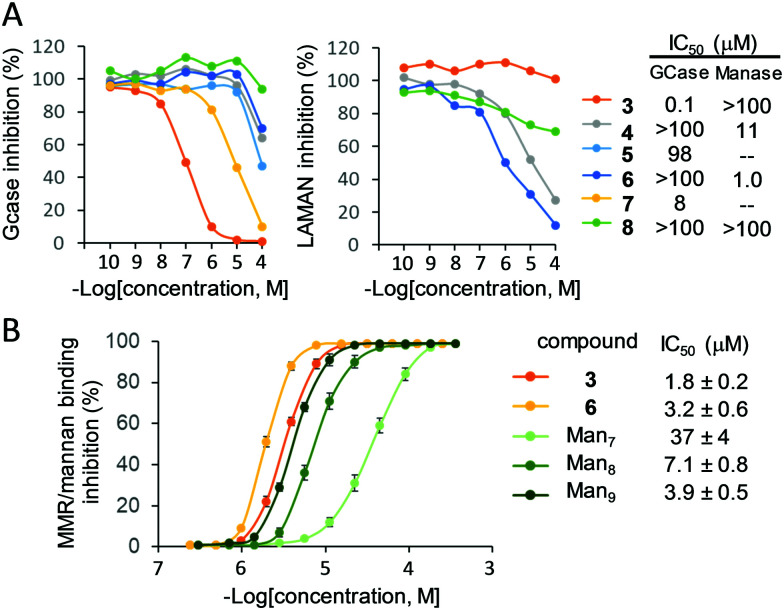
(A) Inhibition plots and of IC_50_ values for 3–8 against GCase and LAMAN in cell lysates (human fibroblasts). (B) ELLA plots and IC_50_ values for the inhibition of recombinant human MMR binding to yeast mannan by increasing concentrations of the multimannosides 3 and 6 in comparison with the natural high-mannose oligosaccharides Man_7_, Man_8_ and Man_9_. Data represent mean values of three independent experiments ± SD.

The fact that the multivalent glycosides 3–8 behave as inhibitors, instead of substrates, of biologically relevant enzymes is remarkable. To get some clues on the mechanism, we further carried out glycosidase/lectin competitive assays with the commercial proteins in the absence and presence of inhibitors with known binding modes (Fig. S21, ESI†).^25^ The results strongly suggested that aglycone and surface binding sites play a critical role in multivalent enzyme inhibition. Considering that bovine liver β-glucosidase and jack bean α-mannosidase share similar catalytic site architectures than GCase (glycosyl hydrolase family GH1) and LAMAN (GH38), respectively,^26^ the implication of non-glycone enzyme regions in glycocluster binding can also be inferred for the later. In any case, much work is necessary to fully ascertain the molecular basis of the observed selectivities.

The MMR recognition capabilities of the better performing inhibitors 3 (for GCase) and 6 (for LAMAN) were next validated by a modified ELLA protocol against the high mannose-type oligosaccharides Man_7_, Man_8_ and Man_9_ (Fig. 3B; see the ESI† for structure representations), the putative ligands of this lectin.^27^ The results corroborated the remarkable enzyme/lectin dual-targeting properties of both prototypes, with MMR binding efficiencies surpassing that of Man_9_. Of note, differently from 3 and 6, Man_7_, Man_8_ and Man_9_ were substrates of the GH38 jack bean Manase in a control experiment. Since binding to the MMR inherently elicits macrophage uptake, these compounds epitomize the first examples of intrinsically site-specific, self-deliverable glycosidase regulators. We believe that this notion represents a new paradigm in multivalent glycoligand design, where the multiconjugation of a glycoside motif serves to assemble glycosidase modulators that become their own delivery agents. Nonetheless, exploiting lectin/glycosidase cross-talk behaviors elicited by multivalency should be applicable to other biomedically important problems were the regulation of a glycosidase in a given organ or tissue represents a potential therapy, ranging from congenital defects of glycosylation to metabolic disorders or cancer.

The authors thank MINECO and MCIU/AEI (SAF2016-76083-R and RTI2018-097609-B-C21), the Junta de Andalucía (FQM2012-1467), the European Regional Development Funds (FEDER and FSE, UE), CNRS, Université Grenoble Alpes, ICMG FR 2607, the French ANR project Glyco@Alps (ANR-15-IDEX-02), Labex ARCANE, CBH-EUR-GS (ANR-17-EURE-0003), the COST action GLYCONanoPROBES (CM18132) and the JSPS KAKENHI Grant 17K10051 for financial support. We acknowledge the CSIC (URICI) for supporting open access publication and the CITIUS (Univ. Seville) for technical support. O. R. acknowledges the European Research Council Consolidator Grant “LEGO” (647938) for D. G. M. G.-C. is a FPI fellow.

## Conflicts of interest

There are no conflicts to declare.

## Supplementary Material

CC-055-C9CC06376E-s001
