# Supplementary material for: Multivalent glycoligands with lectin/enzyme dual specificity: self-deliverable glycosidase regulators
Source: Chem Commun (Camb). 2019 Oct 1;55(85):12845–8. doi: 10.1039/c9cc06376e (PMC12954580; doi:10.1039/c9cc06376e)
Supplement: CC-055-C9CC06376E-s001 [file CC-055-C9CC06376E-s001.pdf]

Electronic Supplementary Material (ESI) for ChemComm.
This journal is © The Royal Society of Chemistry 2019

Multivalent glycoligands with lectin/enzyme dual specificity: self-deliverable glycosidase regulators

Manuel González-Cuesta,[a] David Goyard,[b] Eiji Nanba,[c] Katsumi Higaki,[c] José M. García Fernández,*[d] Olivier Renaudet*[b,e] and Carmen Ortiz Mellet*[a]

List of contents

General methods. S2

Inhibition studies with commercial enzymes. S2

Human enzyme assay. S3

Enzyme-linked lectin assay (ELLA). S3

Two-site ELLA (sandwich assay). S4

Two-site competitive lectin–glycosidase ELLA. S5

Two-site competitive lectin–glycosidase ELLA in the presence of reference inhibitors. S5

Determination of recombinant human MMR (rhMMR) binding affinities. S6

Synthesis of new compounds. S7

HPLC Chromatograms. S11

Copies of the NMR Spectra of the new compounds**.** S13

Inhibition constants values for **3**-**8** against commercial glycosidases. S16

Lineweaver-Burk / Dixon plots for glycosidase inhibition. S16

Enzyme-linked lectin assay (ELLA) and two-site ELLA data S19

Inhibition plots for **3**-**8** against lysosomal α-glucosidase and β-mannosidase in cell lysates S20

Lectin/glycosidase competitive assays S20

General Methods. All commercial chemicals were of reagent grade and were used without further purification. 6-Azidohexyl α-D-manopyranoside (**14**),[1] hepta(2,3-di-*O*-methyl-6-*O*-propargyl)cyclomaltoheptaose (**15**),[2] heptakis (2,6-di-O-propargyl)cyclomaltoheptaose (**16**),[3] pentynoic acid succinimide ester (**17**)[4], the tetravalent mannosylated glycocluster **7**[5] and **9**[5] and the azide-functionalized RAFT cyclopeptide **20**[6] were prepared following the previously reported protocols. All protected amino acids, Fmoc-Gly-Sasrin® and Rink Amide MBHA resins were obtained from Advanced ChemTech Europe (Brussels, Belgium), BachemBiochimie SARL (Voisins-Les-Bretonneux, France) and France Biochem S.A. (Meudon, France). For peptides and glycopeptides, analytical RP-HPLC was performed on a Waters alliance 2695 separation module, equipped with a Waters 2489 UV/visible detector. Analyses were carried out at 1.23 mL/min (Interchim UPTISPHERE X-SERIE, C_{18}, 5 µm, 125x3.0 mm) with UV monitoring at 214 nm and 250 nm using a linear A–B gradient (buffer A: 0.09% CF_3CO_2H in water; buffer B: 0.09% CF_3CO_2H in 90% acetonitrile). Preparative HPLC was performed on Waters equipment consisting of a Waters 600 controller and a Waters 2487 Dual Absorbance Detector equipped with a fraction collector. Purifications were carried out at 22.0 mL/min (VP 250x21 mm nucleosil 100-7 C_{18}) with UV monitoring at 214 nm and 250 nm using a linear A–B gradient. Optical rotations were measured at 20 ± 2 °C in 1-dm tubes on a Jasco P-2000 polarimeter. ^{1}H (and ^{13}C NMR) spectra were recorded at 500 (125.7) MHz with Bruker 500 DRX o BrukerAvance III spectrometers and chemical shifts (δ) were reported in parts per million (ppm). 1D TOCSY, 2D COSY, HMQC and HSQC experiments were used to assist on NMR assignments. Thin-layer chromatography (TLC) was carried out on aluminum sheets coated with silica gel 60 F254 Merck with visualization by UV light and by charring with ethanolic 10% H_2SO_4 and 0.1% ninhydrin. Column chromatography was carried out on Silica Gel 60. ESI mass spectra of βCD derivatives were recorded on a Bruker Daltonics Esquire6000™ ion-trap mass spectrometer. ESI mass spectra of peptides and glycopeptides were measured on an Esquire 3000 spectrometer from Bruker or on an Acquity UPLC/MS system from Waters equipped with a SQ Detector 2. MALDI-TOF were performed on a AutoFlex I Bruker after sample pre-treatment in an OligoR3 microcolumn (Applied Biosystems, USA) using 2,5-dihydroxybenzoic acid matrix. HRMS analyses were performed on a Waters Xevo® G2-S QTof at Mass Spectrometry facility, PCN-ICMG, Grenoble. Elemental analyses were carried out at the Instituto de Investigaciones Químicas (Sevilla, Spain).

Inhibition studies with commercial enzymes. Inhibition constant (K_i) values were determined by spectrophotometrically measuring the residual hydrolytic activities of the glycosidases against the respective *p*-nitrophenyl α- or β-D-glycopyranoside or *o*-nitrophenyl β-D-galactopyranoside (for β-galactosidases) in the presence of the tested glycocluster. Each essay

was performed in phosphate buffer or phosphate-citrate buffer (for α- or β-mannosidase) at the optimal pH of each enzyme. The reactions were initiated by addition of enzyme to a solution of the substrate in the absence or presence of various concentrations of inhibitor. The mixture was incubated for 10−30 min at 37 °C, and the reaction was quenched by addition of 1 M Na_2CO_3. Reaction times were appropriate to obtain 10−20% conversion of the substrate to achieve linear rates. The absorbance of the resulting mixture was determined at 405 nm. Approximate value of K_i was determined from the slope of Dixon plots using a fixed concentration of substrate (around the K_m value for the different glycosidase) and various concentrations of inhibitor. Full K_i determinations and enzyme inhibition mode were determined from the slope of Lineweaver-Burk plots and double reciprocal analysis. Representative example of Dixon and Lineweaver-Burk plots are shown hereinafter.

Human Enzyme Assay. Human normal skin fibroblasts were maintained in our laboratory. These cells were cultured in DMEM supplemented with 10% FBS. Human lysosomal glycosidase activity in cell lysate was measured by using 4-methylumbeliferyl (4-MU) glycoside substrates. Briefly, cells in 35-mm dishes were washed with PBS and collected in 200 μL 0.1% Triton X-100 in distilled H_2O. After centrifugation (4,032 cfr for 15 min at 4 °C) to remove insoluble materials, 2 μL of lysates with 4 μL of the substrate solution in 0.1 M citrate buffer (pH 4) was incubated at 37 °C for 60 min and the reaction was terminated by adding 0.2 M glycin-NaOH buffer (pH 10.7). The liberated 4-MU was measured with a fluorescence plate reader (excitation 340 nm; emission 460 nm; Infinite F500, TECAN Japan, Kawasaki, Japan). Protein concentrations were determined using Protein Assay Rapid Kit (WAKO, Tokyo, Japan) and enzyme activity was normalized by protein concentration. For determination of the inhibitory potenty of the glycoclusters, cell lysates in 0.1% Triton X-100 in distilled H_2O from normal skin fibroblasts were used. Lysates were mixed with the corresponding 4-MU substrate in the absence or presence of increasing concentrations of the tested compounds, incubated at 37 °C for 60 min and the reaction was terminated by adding 0.2 M glycin-NaOH buffer (pH 10.7). The liberated 4-MU was measured with a fluorescence plate reader as described above. The inhibition percentage vs compound concentration plots for GCase and LAMAN are depicted in Figure 4A in the manuscript. The analogous plots for lysosomal α-glucosidase and β-mannosidase are depicted in Figure S19 hereinafter.

Enzyme-linked lectin assay (ELLA). Nunc-Inmuno plates (MaxiSorpTM) were coated overnight with yeast mannan (for ConaA) or click lactose-polystyrene glycopolymer[7] (for PNA) at 100 μL

per well diluted from a stock solution of 10 μg·mL^{-1} in 0.01 M phosphate buffer saline (PBS, pH 7.3 containing 0.1 mM Ca^{2+} and 0.1 mM Mn^{2+}) at room temperature. The wells were then washed three times with 300 μL of washing buffer (containing 0.05% (v/v) Tween 20; PBST). The washing procedure was repeated after each of the incubations throughout the assay. The wells were then blocked by incubation with 150 μL per well of 1% BSA/PBS for 1 h at 37 °C and washed again. For determination of horseradish peroxidase labeled Concanavalin A (*Concanavalia einsiformis*) lectin (HRP-ConA) or Peanut agglutinin (*Arachis hypogaea*) lectin (HRP-PNA) binding affinity, the wells were filled with 100 μL of serial dilutions of of the corresponding HRP-labelled lectin from 10^{-1} to 10^{-5} mg·mL^{-1} in PBS, and incubated at 37 °C for 1 h. The plates were washed and 50 μL per well of 2,2'-azinobis-(3-ethylbenzothiazoline-6-sulfonic acid) diammonium salt (ABTS; 0.25 mg·mL^{-1}) in citrate buffer (0.2 M, pH 4.0 with 0.015% H_2O_2) was added. The reaction was stopped after 20 min by adding 50 μL per well of 1 M H_2SO_4 and the absorbance was measured at 405 nm. Blank wells contained citrate-phosphate buffer. The concentration of lectin–enzyme conjugate that displayed an absorbance between 0.8 and 1.0 was used for inhibition experiments.

To carry out the inhibition experiments, each ligand (**3-8**) was added in a serial of two fold dilutions (50 μL per well) in PBS to the lectin-peroxidase conjugate at the desired concentration (60 μL) on Nunclon (Delta) microtiter plates and incubated for 1 h at 37 °C. The above solutions (100 μL) were then transferred to the mannan-coated or lactose polymer-coated microplates, which were incubated for 1 h at 37 °C. The plates were washed and the ABTS substrate was added (50 μL per well). Color development was stopped after 20 min by adding 50 μL per well of 1 M H_2SO_4 and the absorbance was measured. The percent of inhibition was calculated as follows:

$$\text{Inhibition (\%)} = (A_{(\text{no inhibitor})} - A_{(\text{with inhibitor})}) / A_{(\text{no inhibitor})} \times 100$$

Results in triplicate were used for the plotting the inhibition curves for each individual ELLA experiment. Typically, the IC_{50} values (concentration required for 50% inhibition of the ConA-coating mannan binding) obtained from several independently performed tests were in the range of ±12%. Nevertheless, the relative inhibition values calculated from independent series of data were highly reproducible (Figure S19A).

Two-site ELLA (sandwich assay). The mannan-coated Nunc-Inmuno plates (MaxiSorpTM) microtitration plates were incubated with ConA lectin at 100 μL per well of a stock solution of 5

μg·mL^{-1} in 0.01 M phosphate buffer (PBS, pH 7.3) for 2 h at 37 °C. The ligands **3-8** (200 μM) and mannose (5 mM) as a negative control were used as stock solutions in PBS. The ligands were added in serial two- to ten-fold dilutions (50 μL per well) in PBS with the desired horseradish peroxidase-labeled Con A lectin concentration (50 μL per well) on Nunclon (Delta) microtiter plates and incubated at 37 °C. After 1 h, the above solutions (100 μL) were transferred to the mannan-coated microplates, which were incubated for 1 h at 37 °C. The plates were washed with PBS, and 50 μL per well of ABTS (1 mg/4 mL) in citrate-phosphate buffer (0.2 M, pH 4.0 with 0.015% H_2O_2) was added. The reactions were stopped after 20 min by adding 50 μL per well of 1 M H_2SO_4, and the optical density was measured at 410 nm relative to 570 nm (Figure S19B).

Two-site competitive lectin–glycosidase ELLA. The mannan-coated microplates prepared as above described were further coated with ConA or PNA lectin at 100 μL per well of a stock solution of 5 μg·mL^{-1} in 0.01 M phosphate buffer (PBS, pH 7.3) for 2 h at 37 °C. The ligands **3**-**8** (72 μM stock solutions in PBS except for **5** and **7**, for which 125 μM stock solutions were used) were added (50 μL per well) on Nunclon (Delta) microtiter plates. At this concentration, a classical two-site ELLA provided optical density values of 0.3-0.6 (absorbance units; A.U.), which were normalized in all cases at 100% cross linking for the lectin–glycosidase competition experiments. The corresponding glycosidase (bovine liver β-glucosidase or Jack bean α-mannosidase) in serial twofold dilutions (50 μL per well) from a stock solution of 40 U per mL in PBS and the desired HRP–ConA lectin concentration (50 μL per well) were then added on Nunclon (Delta) microtiter plates and incubated at 37 °C. The plates were washed with PBS, and 50 μL per well of ABTS (1 mg/4 mL) in citrate-phosphate buffer (0.2 M, pH 4.0 with 0.015% H_2O_2) was added. The reactions were stopped after 30 min by adding 50 μL per well of 1M H_2SO_4, and the optical density was measured at 410 nm relative to 570 nm (Figure S21A). Control experiments were conducted to confirm that the enzyme itself did not interact with ConA and that it retained its catalytic activity under the conditions of the assay. Form this assay, the lowest concentration of the enzyme provoking a reduction in the HRP-ConA—ConA crosslinking abilities of the conjugates below 30% of the initial value in the absence of the enzyme (10 U·mL^{-1} for Glcase; 15 U·mL^{-1} for Manase) was selected for the next competitive assay in the presence of reference inhibitors.

Two-site competitive lectin–glycosidase ELLA in the presence of reference inhibitors. To get information about the involvement of the catalytic and non-glycone sites in the binding of the enzyme (bovine liver β-glucosidase or Jack bean α-mannosidase) to the polymannosylated

conjugates **3**-**8** the above protocol was repeated using in the last incubation step a solution of the desired HRP-ConA lectin concentration (50 μL per well) that contained inhibitors targeting the glycone, glycone/aglycone and surface sites (**9**-**13**; 500 μM; Figure S21B). Control experiments demonstrated that neither of the inhibitors affected ConA/HRP-ConA crosslinking in the absence of the enzyme.

Determination of recombinant human MMR (rhMMR) binding affinities. The wells of mannan-coated microplates, prepared as above described, were filled with 100 μL of serial dilutions of rhMMR from a 10 $\mu g \cdot mL^{-1}$ stock solution in PBS (pH 7.3 containing 0.1 mM Ca^{2+} and 0.1 mM Mn^{2+}), and incubated at 37 °C for 1 h. The plates were washed three times with PBST as described above and 100 μL of a solution of biotinylated anti-human MMR antibody (0.2 $\mu g \cdot mL^{-1}$, R&D Systems) in PBS was added in each well, and the plates were further incubated for 1 h at 37 °C. The complex NeutrAvidin-biotinylated HRP was preformed separately by successively adding to Tris buffer (9.6 mL, 50 mM, pH 7.6) a solution of NeutrAvidin (100 $\mu g \cdot mL^{-1}$ in Tris buffer, 1.2 mL, Thermo Scientific) and a solution of biotin-conjugated HRP (25 $\mu g \cdot mL^{-1}$ in Tris buffer, 1.2 mL, Thermo Scientific). The mixture was shaken for 30 min at RT and the solution was immediately transferred into the plates (60 μL/well). After 1 h at 37 °C, these plates were washed twice with Tris (250 μL/well) and ABTS (0.25 $mg \cdot mL^{-1}$, 50 μL/well) in citrate buffer (0.2 M, pH 4.0 with 0.015% H_2O_2) was added. After 5 min at RT, the optical density was measured at 415 nm. Control wells were processed with anti-human MMR antibody as well as NeutrAvidin-biotinylated HRP. The concentration of rhMMR that displayed an absorbance between 0.8 and 1.0 was used for inhibition experiments. For the competitive lectin-binding inhibition experiment, the multimannosides **3**, **6** or the high-mannose oligosaccharides Man_7, Man_8 or Man_9 (see structures hereinafter) used as reference ligands, in a serial of 2-fold dilutions (60 μL per well) in HEPES buffer (20 mM, pH 7.4), were mixed with 60 μL of the appropriate rhMMR concentration in PBS buffer on Nunclon (Delta) microtitre plates and incubated for 1 h at 37 °C. The above solutions (100 μL) were then transferred to the mannan-coated titer plates, which were incubated for 1 h at 37 °C. The plates were washed and the solution of biotinylated anti-human MMR antibody in PBS (100 μL) was added in each well, and the plates were further incubated for 1 h at 37 °C. The NeutrAvidin solution was then transferred into the plates (60 mL/well). After 1 h at 37 °C, these plates were washed twice with Tris (250 μL/well) and ABTS was added (50 μL/well). Optical density at 415 nm was determined after 5 min. The percent of inhibition was calculated as above indicated for a typical ELLA experiment.

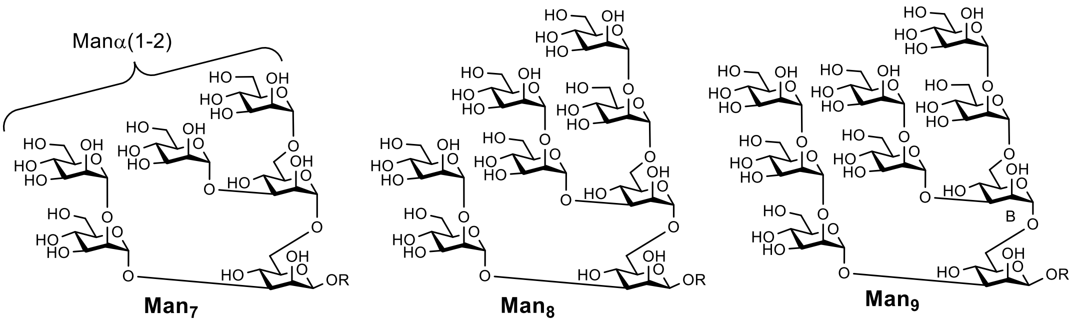

Synthesis of new compounds

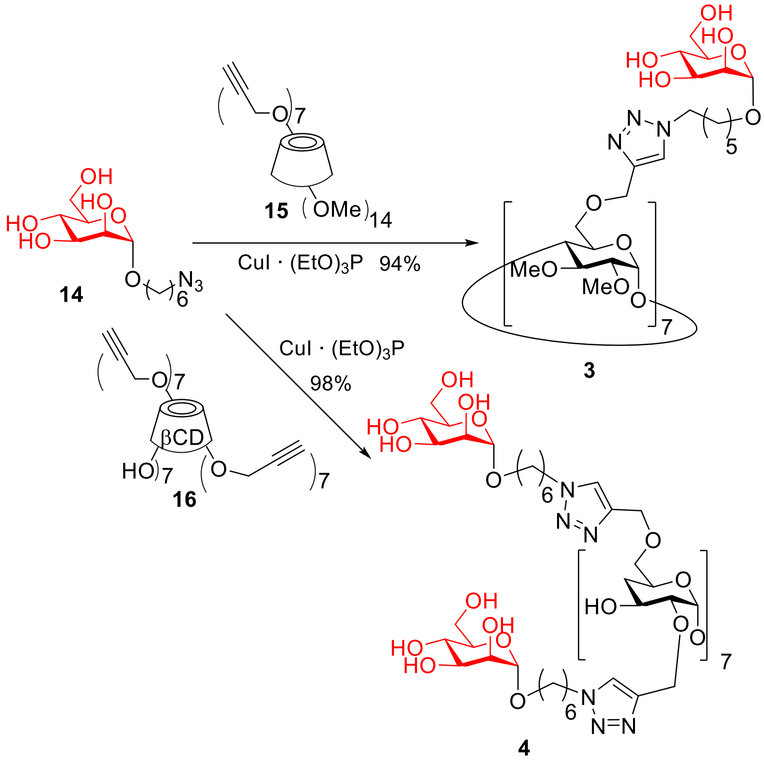

Heptakis[2,3-di-*O*-methyl-6-(α-D-mannopyranosyloxy)hexyl-1*H*-1,2,3-triazol-4-ylmethyl]cyclomaltoheptaose (3). To a solution containing the 6-azidohexyl derivative **14**[1] (0.16 mmol) and the heptapropargylated βCD derivative **15**[2] (30 mg, 18.9 μmol) in acetone-MeOH (2:1, 20 mL), CuI·(EtO)$_3$P (5 mg, 13.0 μmol) and DIPEA (22 μL, 0.13 mmol) were added and the mixture was refluxed overnight for 24 h. The solvents were evaporated and the resulting residue was purified by column chromatography (12:6:1→6:3:1 MeCN-H_2O-NH_4OH) to give **3**. Yield: 113 mg (94%). R_f 0.31(6:3:1 MeCN-H_2O-NH_4OH). $[\alpha]_D$ +77.5 (*c* 1.0, H_2O). ^{1}H NMR (500 MHz, CD_3OD, 333 K) δ 7.95 (s, 7 H, =CH), 5.10 (bs, 7 H, H-1), 4.75 (s, 7 H, H-1_{Man}), 4.66-4.58 (m, 14 H, OCH_2), 4.39 (t, 14 H, $^3J_{H,H}$ = 7.1 Hz, H-1_{Hex}), 3.98 (bs, 7 H, H-5), 3.84 (dd, 7 H, $J_{6a,6b}$ = 11.8 Hz, $J_{5,6a}$ = 2.6 Hz, H-$6a_{Man}$), 3.81 (m, 7 H, H-2_{Man}), 3.74 (dd, 7 H, $J_{5,6b}$ = 5.6 Hz, H-$6b_{Man}$), 3.72-3.70 (m, 14 H, H-3_{Man}, H-6_{Hex}), 3.65 (t, 7 H, $J_{3,4}$ = $J_{4,5}$ = 9.6 Hz, H-4_{Man}), 3.64 (s, 21 H, OMe), 3.64 (m, 7 H, H-4), 3.56-3.52 (m, 14 H, H-3, H-5_{Man}), 3.51 (s, 21 H, OMe), 3.46-3.41 (m, 7 H, H-6_{Hex}), 3.14 (dd, 7 H, $J_{2,3}$ = 9.4 Hz,

$J_{1,2}$ = 3.2 Hz, H-2), 1.95-1.90 (m, 14 H, H-2_{Hex}), 1.63-1.58 (m, 14 H, H-5_{Hex}), 1.48-1.44 (m, 14 H, H-4_{Hex}), 1.41-1.36 (m, 14 H, H-3_{Hex}). ^{13}C NMR (125.7 MHz, CD_3OD, 313 K) δ 146.1 (C-4 triazole), 125.1 (C-5 triazole), 101.6 (C-1_{Man}), 99.7 (C-1), 83.4 (C-3), 83.3 (C-2), 80.9 (C-4), 74.6 (C-5_{Man}), 72.8 (C-3_{Man}), 72.5 (C-5), 72.3 (C-2_{Man}), 70.7 (C-6), 68.9 (C-4_{Man}), 68.5 (C-6_{Hex}), 65.4 (OCH_2-triazole), 63.1 (C-6_{Man}), 61.8, 58.9 (OMe), 51.4 (C-1_{Hex}), 31.3 (C-2_{Hex}), 30.4 (C-5_{Hex}), 27.4 (C-3_{Hex}), 26.8 (C-4_{Hex}). ESIMS: *m/z* 1267.4 ([M + 3 Na]$^{3+}$), 1889.9 ([M + 2 Na]$^{2+}$). Anal. Calcd for $C_{161}H_{273}N_{21}O_{77}$: C, 51.77; H, 7.37; N, 7.88. Found: C, 51.39, H, 7.11; N, 7.56.

Heptakis[2,6-di-*O*-(α-D-mannopyranosyloxyhexyl-1*H*-1,2,3-triazol-4-yl)methyl]cyclomaltoheptaose (4). To a solution containing the 6-azidohexyl derivative **14**[1] (107 μmol) and the tetradecapropargylated βCD derivative **16**[3] (15 mg, 88.9 μmol) in acetone-MeOH (1:1, 10 mL), CuI·$(EtO)_3P$ (13.0 μmol) and DIPEA (0.11 mmol) were added and the mixture was refluxed overnight. The solvents were evaporated and the resulting residue was purified by column chromatography (3:2:2 MeCN-H_2O-NH_4OH) to give **4**. Yield: 52.4 mg (98%). R_f 0.39 (3:2:2 CH_3CN:H_2O:NH_4OH). $[\alpha]_D$ +57.9 (*c* 1.0, H_2O). ^{1}H NMR (500 MHz, D_2O, 333 K) δ 8.41 (s, 7 H, =CH), 8.23 (s, 7 H, =CH), 5.30 (d, 7 H, $^2J_{H,H}$ = 18.6 Hz, OCH_2), 5.27 (bs, 7 H, H-1), 5.18-5.17 (m, 21 H, H-1_{Man}, OCH_2), 4.94-4.84 (m, 14 H, OCH_2), 4.78 (t, 14 H, $^3J_{H,H}$ = 7.0 Hz, H-1_{Hex}), 4.65 (t, 14 H, $^3J_{H,H}$ = 7.0 Hz, H-1_{Hex}), 4.27-4.26 (m, 14 H, H-2_{Man}), 4.21 (dd, 7 H, $J_{6a,6b}$ = 12.3 Hz, $J_{5,6a}$ = 2.5 Hz, H-$6a_{Man}$), 4.20 (dd, 7 H, $J_{6a,6b}$ = 12.2 Hz, $J_{5,6a}$ = 2.4 Hz, H-$6a_{Man}$), 4.16-4.12 (m, 28 H, H-$6b_{Man}$, H-3_{Man}), 4.06 (t, 7 H, $J_{3,4}$ = $J_{4,5}$ = 9.7 Hz, H-4_{Man}), 4.05 (t, 7 H, $J_{3,4}$ = $J_{4,5}$ = 9.7 Hz, H-4_{Man}), 4.02-3.98 (m, 14 H, H-6_{Hex}), 3.96-3.89 (m, 21 H, H-5_{Man}, H-2), 3.84-3.76 (m, 14 H, H-6_{Hex}), 2.24 (q, 14 H, $^3J_{H,H}$ = 6.9 Hz, H-2_{Hex}), 2.16 (q, 14 H, $^3J_{H,H}$ = 6.9 Hz, H-2_{Hex}), 1.91-1.85 (m, 28 H, H-5_{Hex}), 1.69-1.56 (m, 56 H, H-4_{Hex}, H-3_{Hex}). ^{13}C NMR (125.7 MHz, D_2O, 333K) δ 144.7, 144.1 (C-4 triazole), 125.5, 124.8 (C-5 triazole), 100.9 (C-1), 100.3, 100.2 (C-1_{Man}), 82.6 (C-3), 79.9 (C-2), 73.3 (C-5_{Man}), 71.4, 71.3 (C-3_{Man}), 71.1 (C-5), 70.8, 70.7 (C-2_{Man}), 69.3 (C-6), 68.1, 68.0 (C-6_{Hex}), 67.4, 67.3 (C-4_{Man}), 65.1, 64.2 (OCH_2-triazole), 61.5 (C-6_{Man}), 50.8, 50.6 (C-1_{Hex}), 29.8 (C-2_{Hex}), 29.0, 28.9 (C-5_{Hex}), 26.0, 25.9 (C-3_{Hex}), 25.3, 25.2 (C-4_{Hex}). ESIMS: *m/z* 1506.5 ([M + 4 Na]$^{4+}$), 2003.4 ([M + 3 Na]$^{3+}$). Anal. Calcd for $C_{252}H_{420}N_{42}O_{119}$: C, 50.94; H, 7.12; N, 9.90. Found: C, 50.57, H 6.801; N, 9.54.

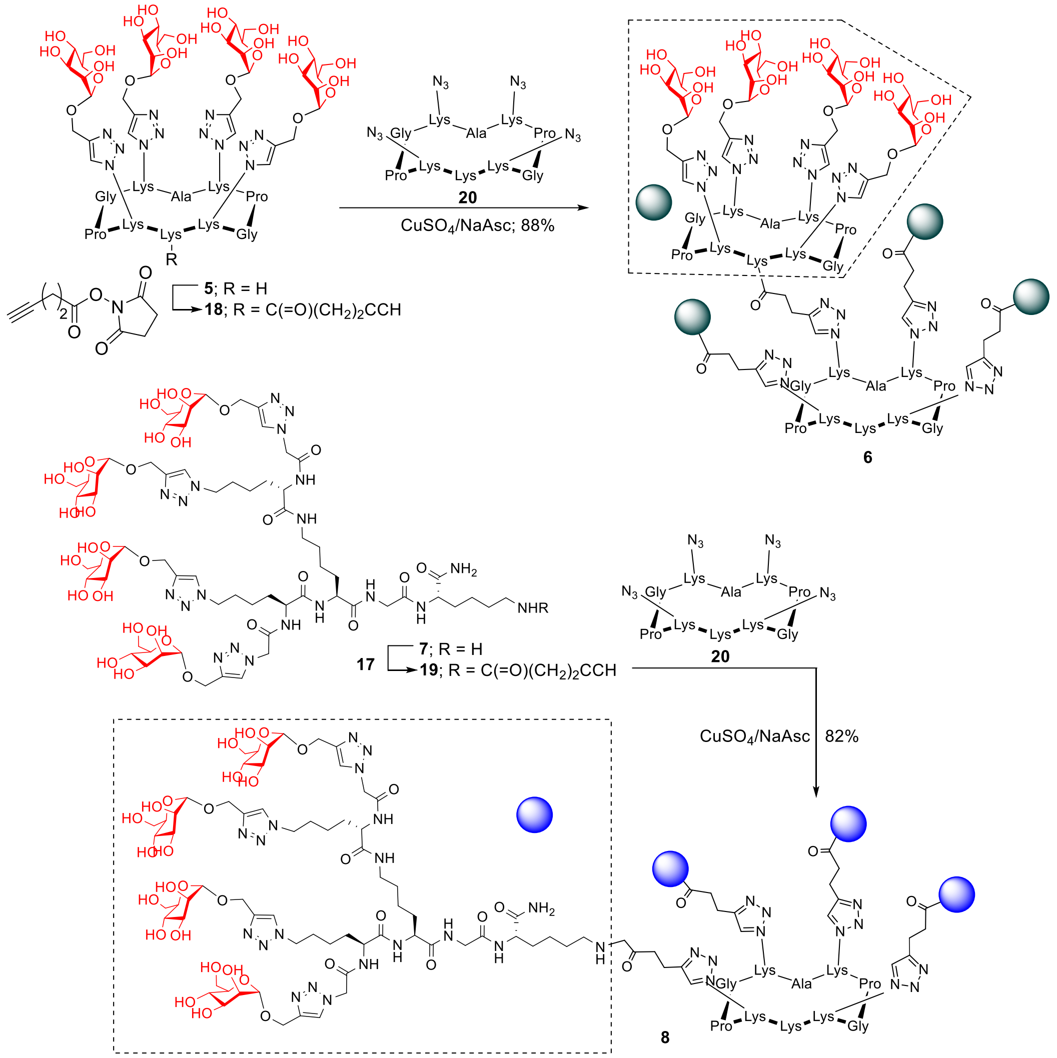

General procedure A for the introduction of the alkyne-armed group on the free lysine residue of tetravalent glycoclusters (18 and 19). The tetravalent RAFT cyclopeptide derivative (**5** or **7**, 1 eq.) was dissolved in dry DMF (1 mL), DIPEA was added to reach pH ≈ 9-10 (c.a. 20 µL) and then pentynoic acid succinimide ester (1.5 eq.) was incorporated. The reaction mixture was stirred at RT for 1 h, after which RP-HPLC showed completion of the reaction. The mixture was concentrated under reduced pressure, dissolved in the minimum volume of methanol (c.a. 200 µL) and precipitated with diethyl ether. The white precipitate was dissolved in water, lyophilized and used without further purification.

General procedure B for the preparation of mannosylated glycodendrimers 6 and 8. Alkyne-substituted glycocluster (**18** or **19**, 4.4 eq.) and azide-functionalized scaffold (**20**, 1 eq.) were dissolved in 1 mL of a 1:1 mixture of DMF and PBS buffer (pH 7.5). Separately, a solution of $CuSO_4 \cdot 5H_2O$ (0.5 eq.) and THPTA (1 eq.) in PBS (300 µL) was added to a solution of sodium ascorbate (3 eq.) in PBS (300 µL). This mixture was added to the solution containing the azide

and alkyne which was degassed with argon and stirred at r.t. for 2 hours after which RP-HPLC showed completion of the reaction. Chelex® resin was then added to the reaction mixture which was stirred for 45 minutes. The resin was filtered off, rinsed with water and the filtrate purified by semi-preparative RP-HPLC. Fractions containing the product were combined and lyophilized.

Alkyne-functionalized tetravalent glycocluster (cyclopeptide core, 18). Compound **18** was prepared according general procedure **A** starting from **5** (32 mg, 16 µmol). The title compound was obtained as a white fluffy solid (29 mg, 14 µmol, 87 %). Purity was assessed by analytical RP-HPLC (R_t = 9.41 min (C_{18}, λ = 214 nm, 0-30% B in 15 min).

Alkyne-functionalized tetravalent glycocluster (polylysine core, 19).
Compound **19** was prepared according general procedure **A** starting from **7** (42 mg, 25 µmol). The title compound was obtained as a white fluffy solid (38 mg, 21 µmol, 88 %). Purity was assessed by analytical RP-HPLC (R_t = 10.10 min (C_{18}, λ = 214 nm, 0-20% B in 15 min).

Hexadecavalent glycodendrimer (cyclopeptide peripheral scaffold, 6).
Compound **6** was prepared according general procedure **B** starting from **18** (29 mg, 14 µmol). The crude mixture was purified by semi-preparative RP-HPLC using a gradient of 0-30% B in 15 minutes. The title compound was obtained as a white fluffy solid after lyophilization (23 mg, 2.5 µmol, 77%). MALDI-ToF *m/z* calcd for $C_{399}H_{626}N_{115}O_{150}$ $[M+H]^+$: 9434.1, found 9434.4; RP-HPLC: R_t = 8.74 min (C_{18}, λ = 214 nm, 0-40% B in 15 min).

Hexadecavalent glycodendrimer (polylysine peripheral scaffold, 8).
Compound **6** was prepared according general procedure **B** starting from **18** (38 mg, 21 µmol). The crude mixture was purified by semi-preparative RP-HPLC using a gradient of 0-30% B in 15 minutes. The title compound was obtained as a white fluffy solid after lyophilization (33 mg, 4.0 µmol, 82%). MALDI-ToF *m/z* calcd for $C_{335}H_{534}N_{103}O_{138}$ $[M+H]^+$: 8210.8, found 8209.5; RP-HPLC: R_t = 9.06 min (C_{18}, λ = 214 nm, 0-30% B in 15 min).

Statistical analysis. All data are average values of three truly independent experiments. Statistical analysis was performed using paired Student's t-test. Data were considered significantly different when p was less than 0.05.

HPLC Chromatograms

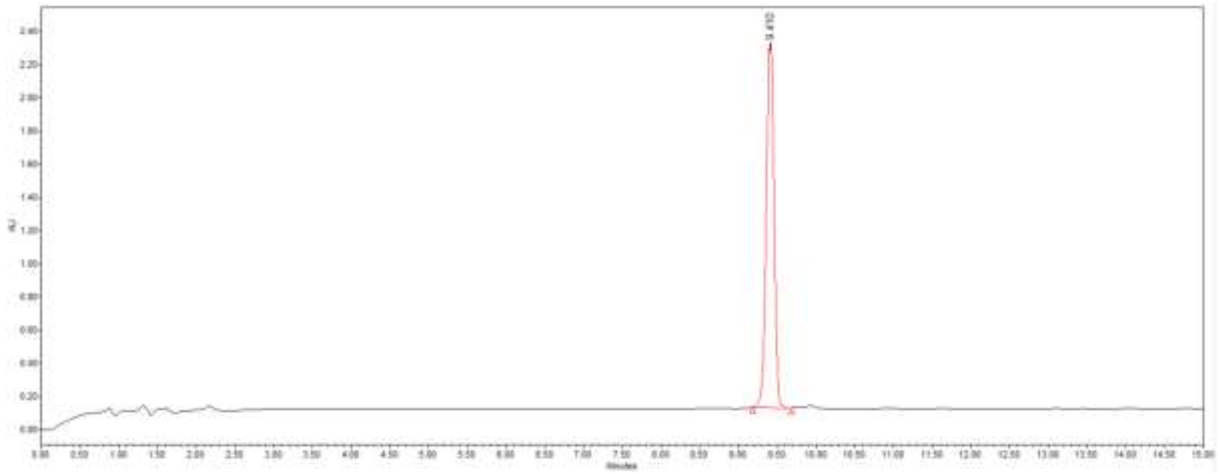

Figure S1. Analytical RP-HPLC spectrum of compound **18.**

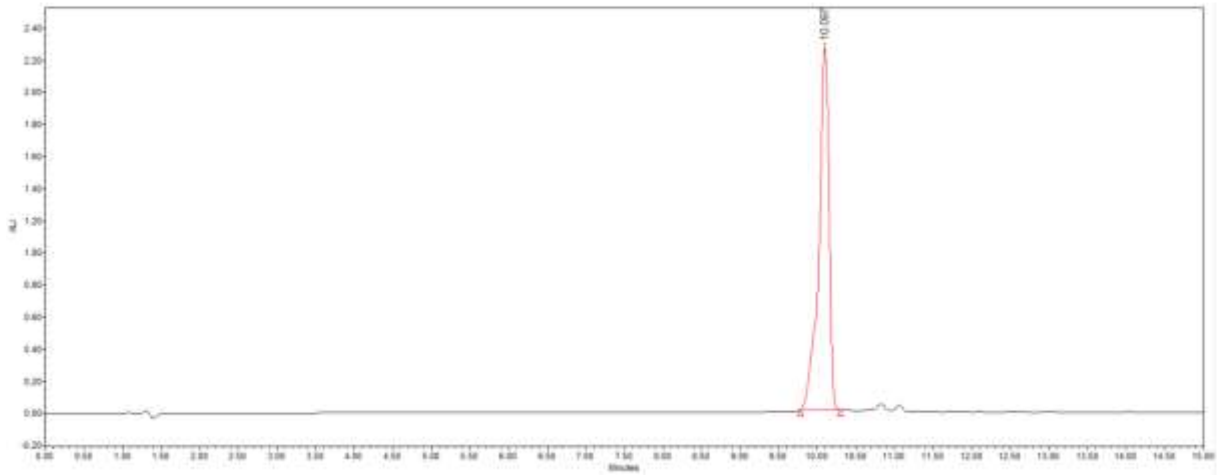

Figure S2. Analytical RP-HPLC spectrum of compound **19.**

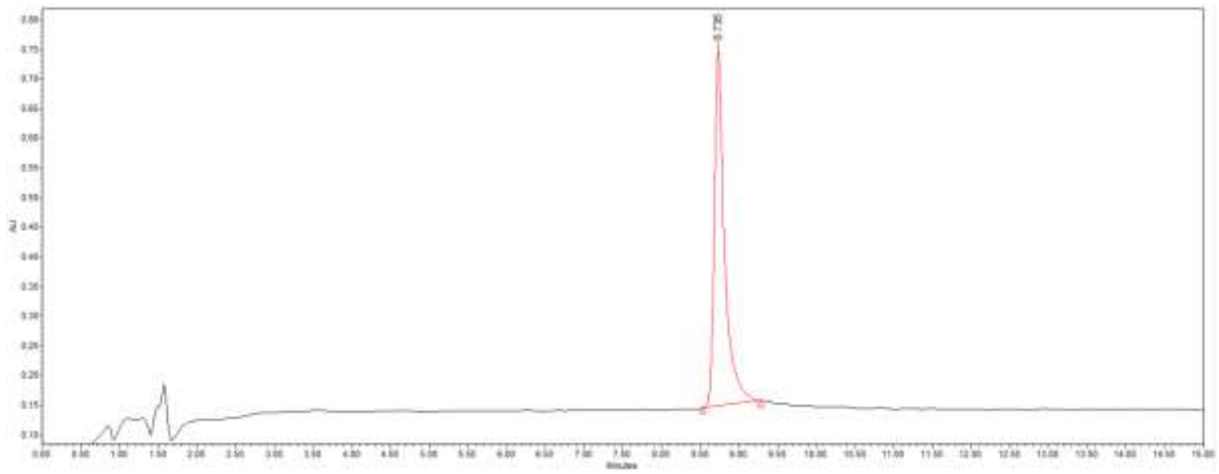

Figure S3. Analytical RP-HPLC spectrum of compound **6.**

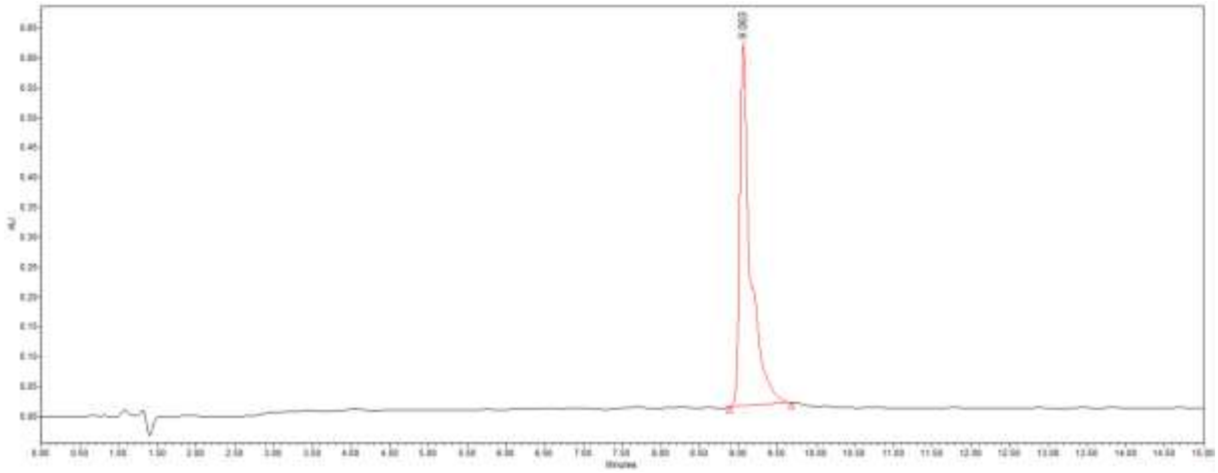

Figure S4. Analytical RP-HPLC spectrum of compound **8.**

NMR Spectra.

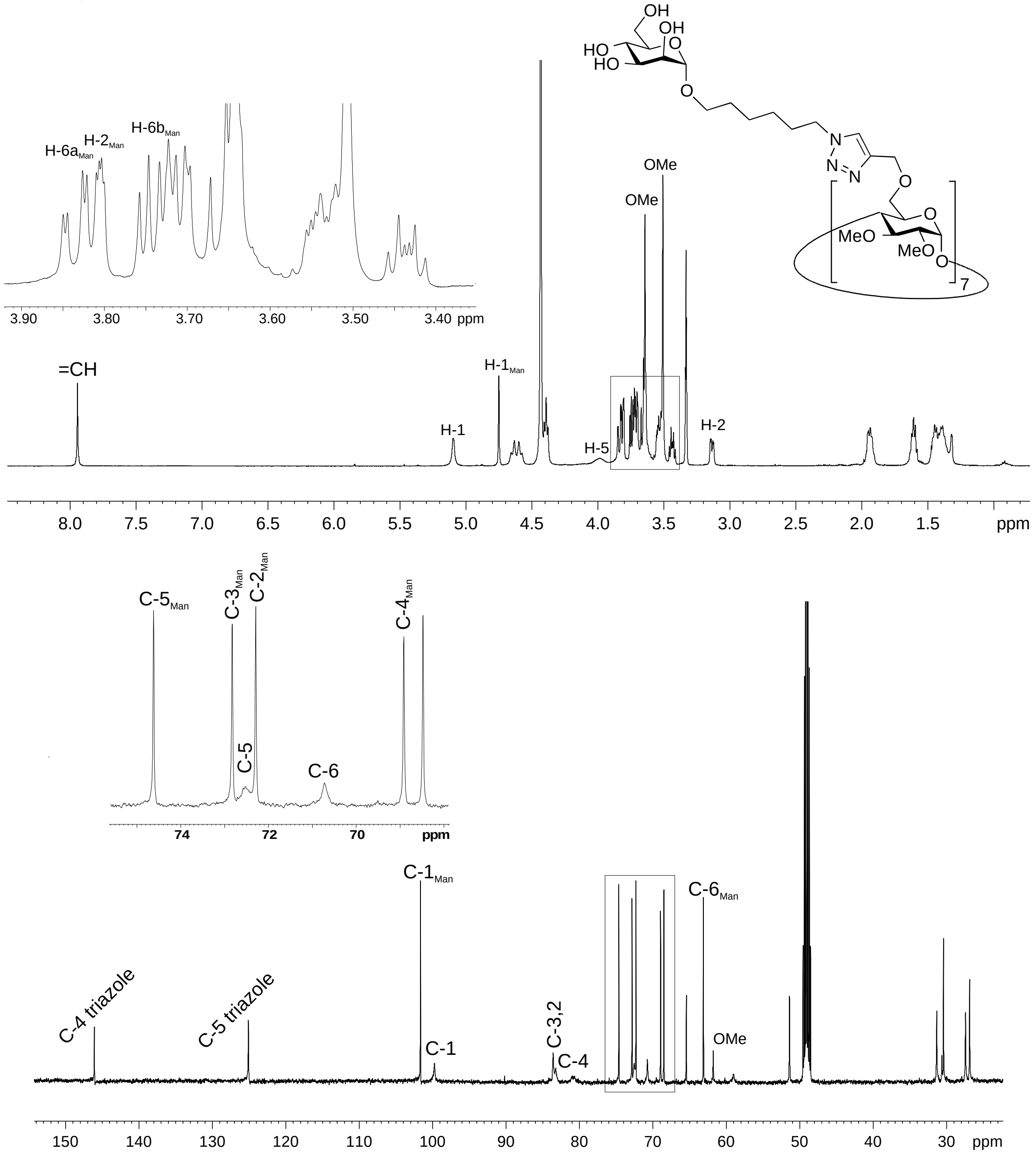

Figura S5. ^{1}H and ^{13}C RMN (500 MHz, 333K, 125.7 MHz, 313K, CD_3OD) of **3**.

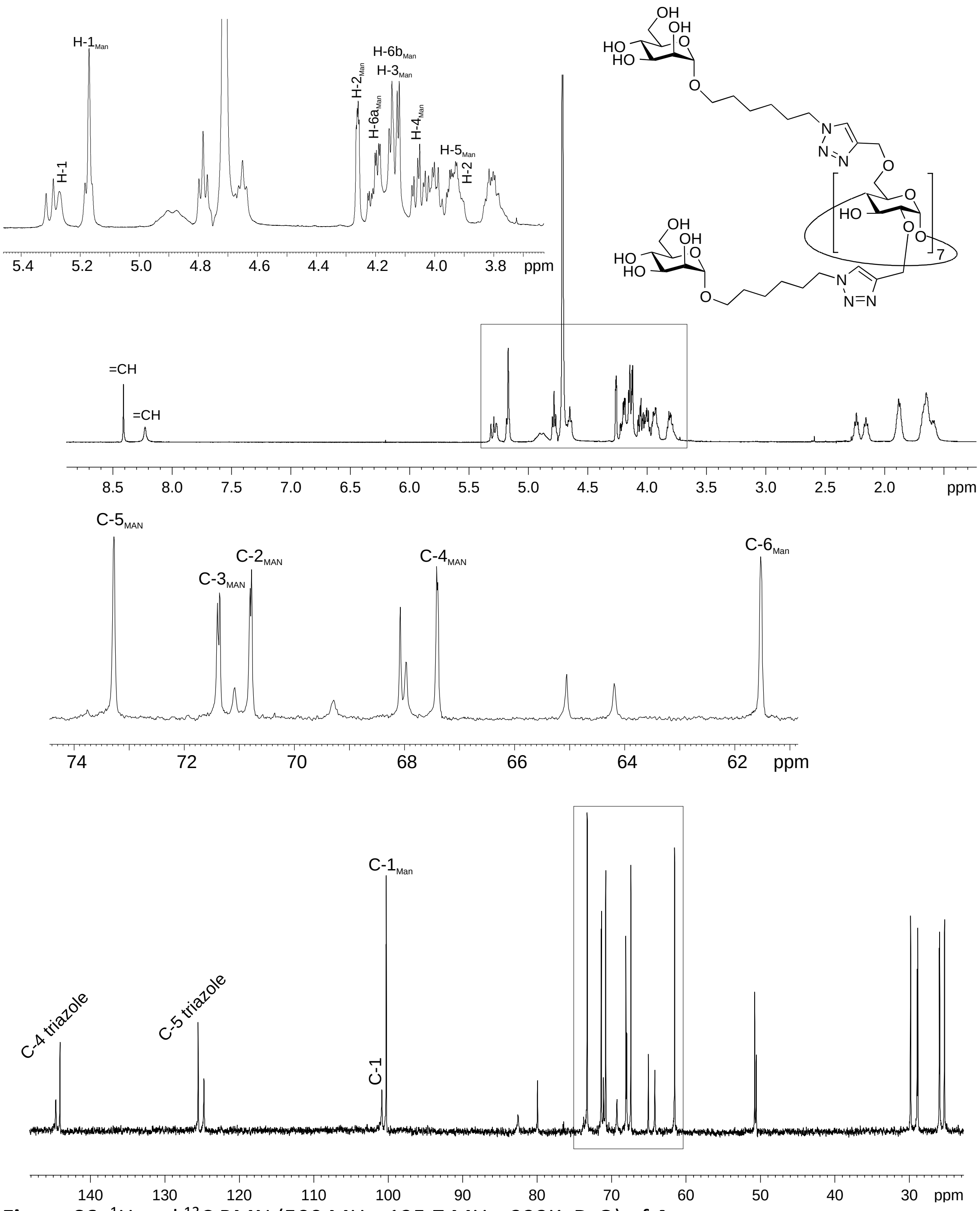

Figura S6. ^{1}H and ^{13}C RMN (500 MHz, 125.7 MHz, 333K, D_2O) of **4**.

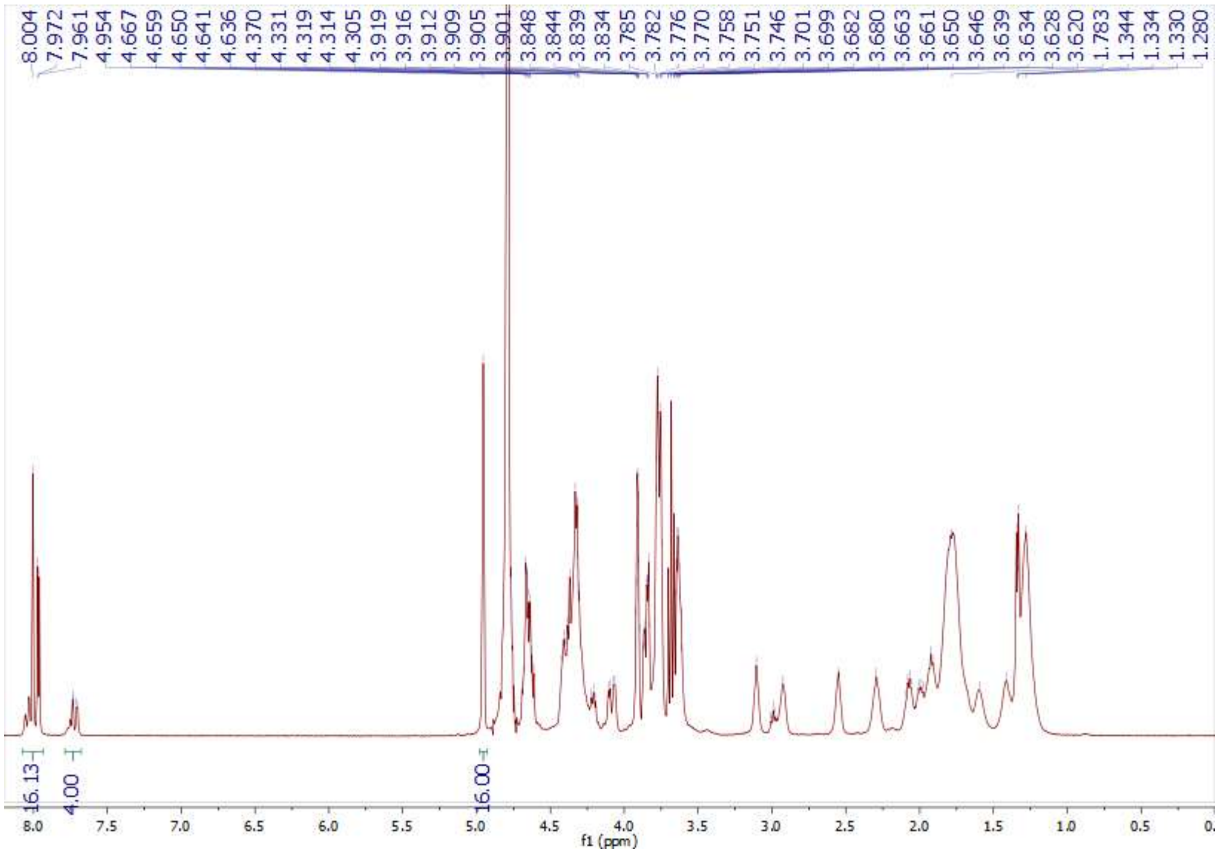

Figure S7. ^{1}H NMR (500 MHz, D_2O) spectrum of **6.**

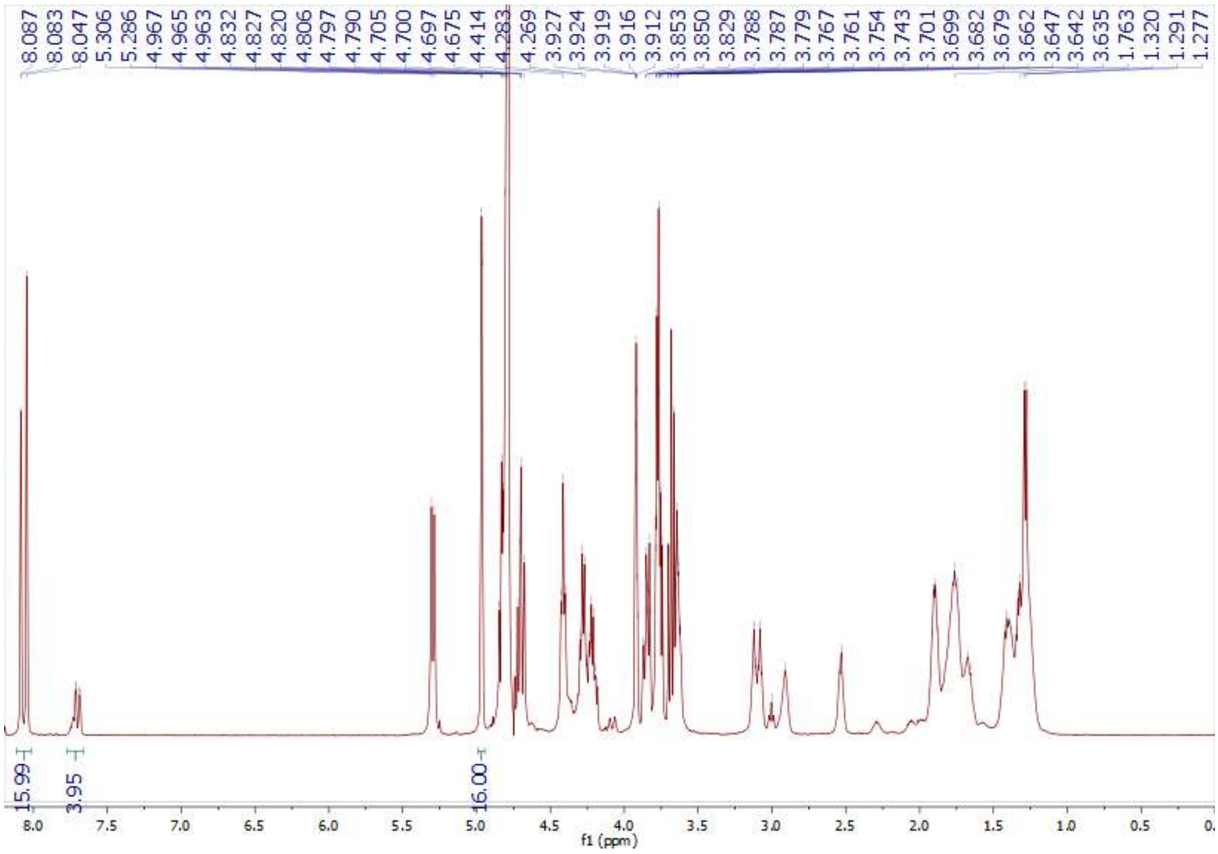

Figure S8. ^{1}H NMR (500 MHz, D_2O) spectrum of compound 8.

Table S1. Inhibitory activity of compounds **3**-**8** against commercial (K_i, μM) glycosidases.

Enzyme	3	4	5	6	7	8
α-glucosidase (*S. cerevisiae*)	129 ± 13	300 ± 30	n.i.	n.i.	n.i.	n.i.
β-glucosidase (*bovine liver*)	7.3 ±0.7	n.i.	327 ± 34	n.i.	65 ± 7	n.i.
α-galactosidase (*green coffee bean*)	n.i.	n.i.	n.i.	n.i.	n.i.	n.i.
β-galactosidase (*E. coli*)	n.i.	n.i.	n.i.	n.i.	n.i.	201 ± 21
α-mannosidase (*jack beans*)	451 ±45	118 ±12	n.i.	32 ± 2	n.i.	86 ± 9

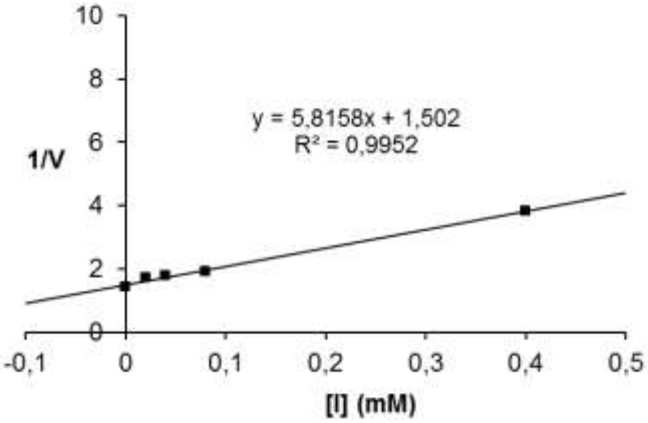

Figure S9. Dixon Plot for K_i determination (129 ± 13 μM) of **3** against *S. cerevisiae* α-glucosidase.

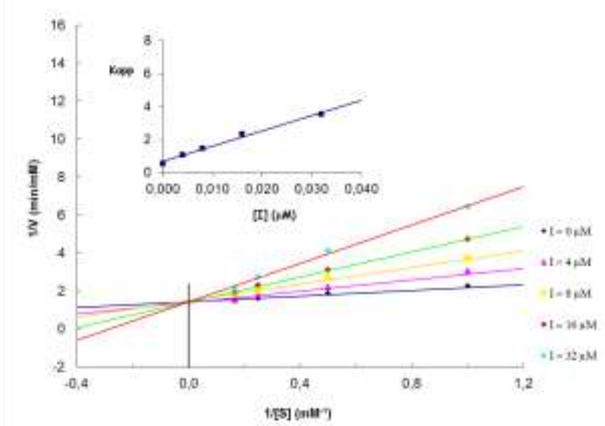

Figure S10. Lineweaver-Burk Plot for K_i determination (7.3 ± 0.7 μM) of **3** against *bovine liver* β-glucosidase.

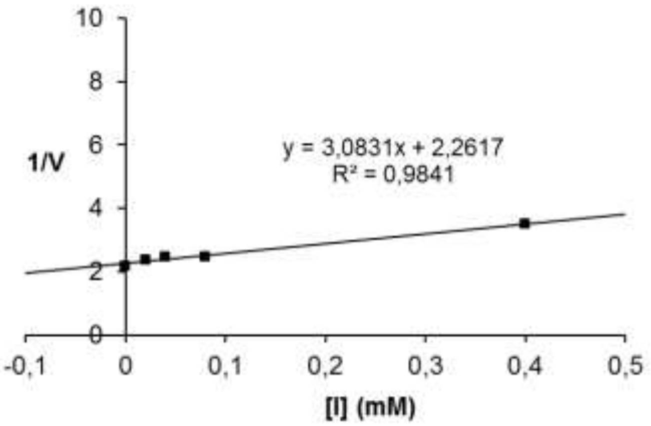

Figure S11. Dixon Plot for K_i determination (7.3 ± 0.7 μM) of **3** against *jack beans α*-mannosidase.

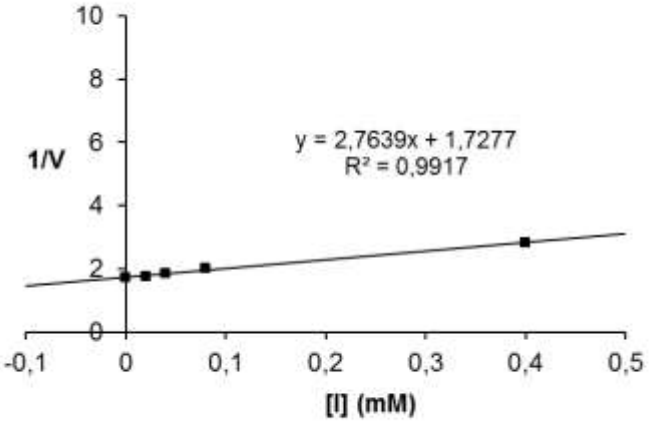

Figure S12. Dixon Plot for K_i determination (300 ±30 μM) of **4** against *S. cerevisiae* α-glucosidase.

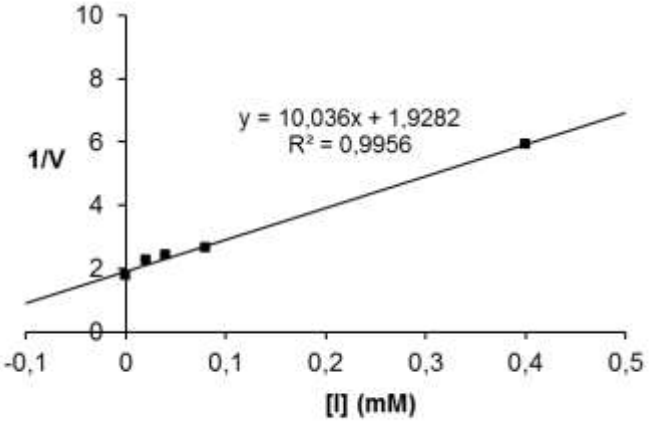

Figure S13. Dixon Plot for K_i determination (118 ±12 μM) of **4** against against *jack beans* α-mannosidase.

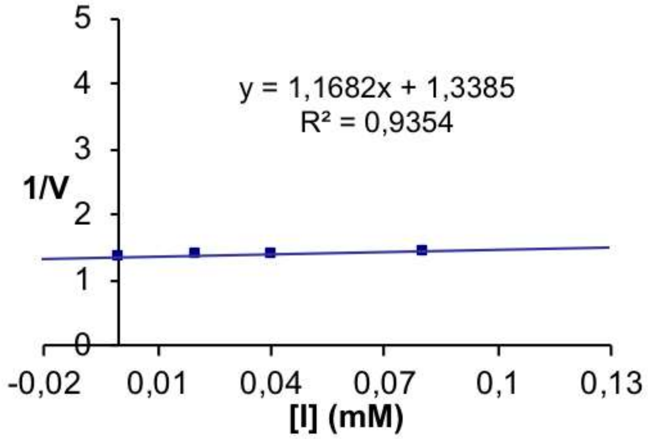

Figure S14. Dixon Plot for K_i determination (327 ± 34 μM) of **5** against *bovine liver* β-glucosidase.

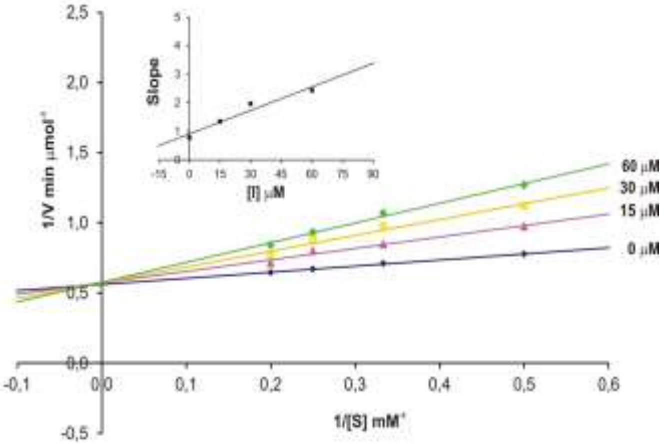

Figure S15. Lineweaver-Burk Plot for K_i determination (32 ± 2 μM) of **6** *jack beans* α-mannosidase.

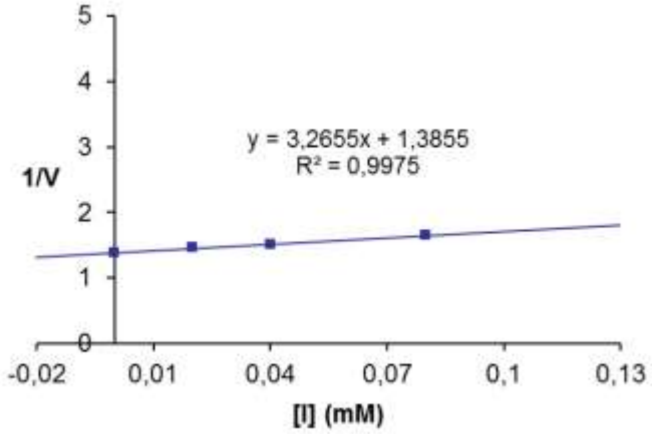

Figure S16. Dixon Plot for K_i determination (201 ± 21 μM) of **8** against *E. coli* β-galactosidase.

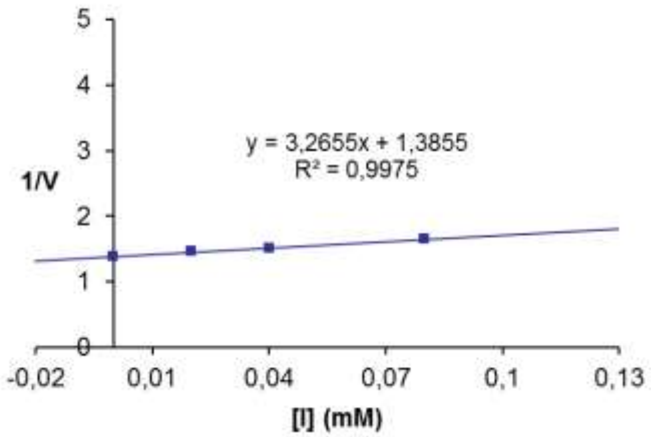

Figure S17. Dixon Plot for K_i determination (201 ± 21 μM) of **8** against *E. coli* β-galactosidase.

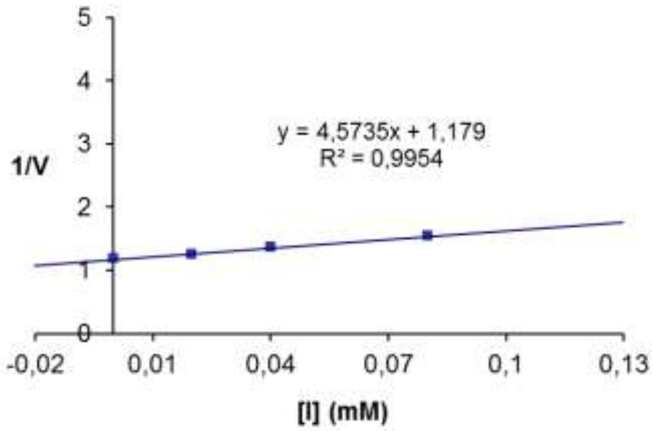

Figure S18. Dixon Plot for K_i determination (86 ± 9 μM) of **8** against *jack beans* α-mannosidase.

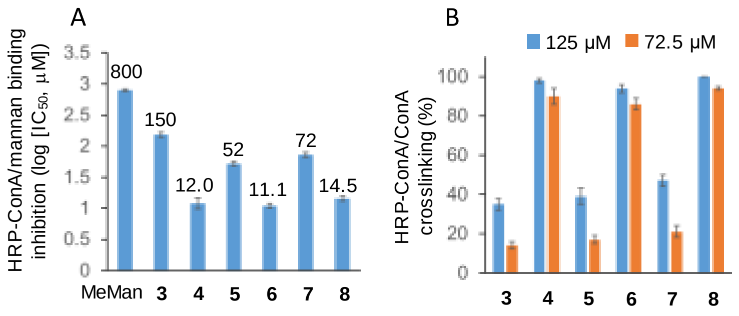

Figure S19. (A) IC_{50} values for HRP-ConA/mannan binding inhibition by compounds **3**-**8** and methyl α-D-mannopyranoside (MeMan) used as monovalent reference as determined by ELLA. (B) Relative HRP-ConA/ConA cross-linking abilities (%) for **3**-**8** (the readout for **8** at 125 μM is taken as 100%) as determined in a two-site ELLA. Bars represent mean values of three independent experiments ± SD.

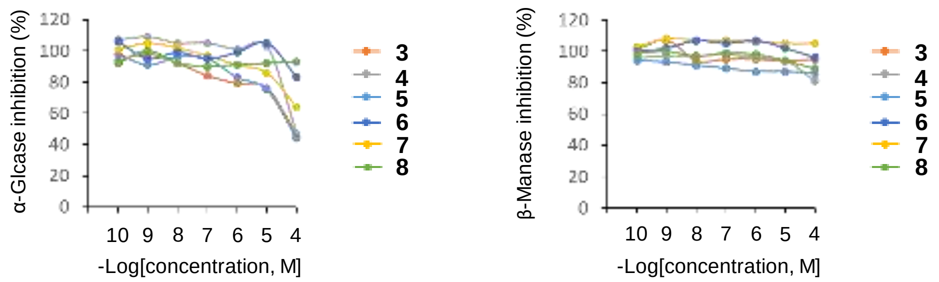

Figure S20. Inhibition plots for **3**-**8** against lysosomal α-glucosidase (α-Glcase, left) and β-mannosidase (β-Manase, right) in cell (human fibroblast) lysates.

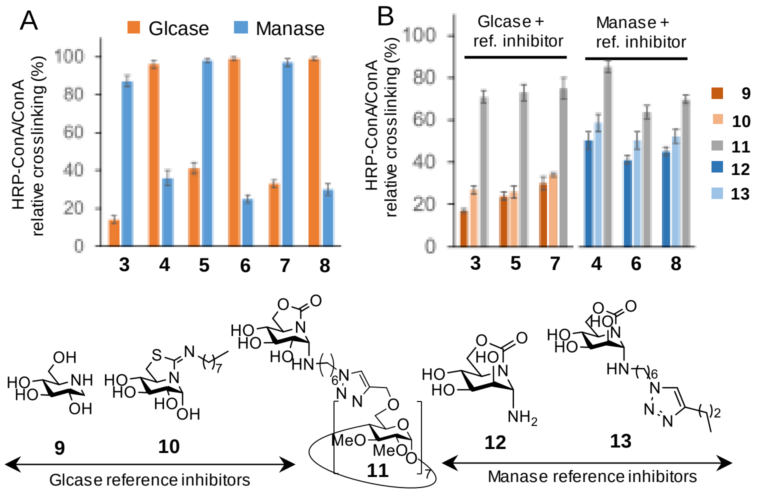

Figure S21. (A) Effect of β-glucosidase (Glcase, bovine liver; 10 U·mL^{-1}) or α-mannosidase (Manase; 15 U·mL^{-1}) in multivalent mannoside-mediated HRP-ConA/ConA cross-linking (**3**-**8**, 72 μM except **5** and **7**, 150 μM); data are given as percentage of cross-linking relative to 100% in the absence of the enzyme. (B) Recovery of the GCase- (for **3**, **5** and **7**) or Manase-depleted (for **4**, **6** and **8**) HRP-ConA/ConA cross-linking abilities promoted by reference inhibitors (**9**-**13**); data are given as percentage of cross-linking recovery relative to 100% in the absence of the inhibitor. Bars represent mean values of three independent experiments ± SD.

[1] E. Kaya, K. Gutsmiedl, M. Vrabel, M. Müller, P. Thumbs, T. Carell, *ChemBioChem*, 2009, **10**, 2858-2861.

[2] F. G. Calvo-Flores, J. Isac-García, F. Hernández-Mateo, F. Pérez-Balderas, A. Calvo-Asín, J. Sánchez-Vaquero, F. Santoyo-González, *Org. Lett.* 2000**, 2**, 2499-2502.

[3] F. Ortega-Caballero, J. J. Giménez-Martínez, A. Vargas-Berenguel, *Org. Lett.* 2003, **5**, 2389-2392.

[4] C. Tiertant, E. Laigre, D. Goyard, O. Renaudet, O. *ACS Omega* 2018, **3**, 14013-14020.

[5] C. Pifferi, D. Goyard, E. Gillon, A. Imberty, O. Renaudet, *ChemPlusChem*, 2017, **82**, 390-398.

[6] I. Bossu, N. Berthet, P. Dumy, O. Renaudet, J. Carbohydr. Chem. 2011, **30**, 458-468.

[7] M. Gómez-García, J. M. Benito, A. P. Butera, C. Ortiz Mellet, J. M. García Fernández, J. L. Jiménez Blanco, *J. Org. Chem.* 2012, **77**, 1273-1288.
